# Evaluation of Novel 3-Hydroxyflavone Analogues as HDAC Inhibitors against Colorectal Cancer

**DOI:** 10.1155/2018/4751806

**Published:** 2018-12-27

**Authors:** Subhankar Biswas, Neetinkumar D. Reddy, B. S. Jayashree, C. Mallikarjuna Rao

**Affiliations:** ^1^Department of Pharmacology, Manipal College of Pharmaceutical Sciences, Manipal Academy of Higher Education, Manipal 576104, Karnataka, India; ^2^Department of Pharmaceutical Chemistry, Manipal College of Pharmaceutical Sciences, Manipal Academy of Higher Education, Manipal 576104, Karnataka, India

## Abstract

Alteration of epigenetic enzymes is associated with the pathophysiology of colon cancer with an overexpression of histone deacetylase 8 (HDAC8) enzyme in this tissue. Numerous reports suggest that targeting HDAC8 is a viable strategy for developing new anticancer drugs. Flavonols provide a rich source of molecules that are effective against cancer; however, their clinical use is limited. The present study investigated the potential of quercetin and synthetic 3-hydroxyflavone analogues to inhibit HDAC8 enzyme and evaluated their anticancer property. Synthesis of the analogues was carried out, and cytotoxicity was determined using MTT assay. Nonspecific and specific HDAC enzyme inhibition assays were performed followed by the expression studies of target proteins. Induction of apoptosis was studied through annexin V and caspase 3/7 activation assay. Furthermore, the analogues were assessed against *in vivo* colorectal cancer. Among the synthesized analogues, QMJ-2 and QMJ-5 were cytotoxic against HCT116 cells with an IC_50_ value of 68 ± 2.3 and 27.4 ± 1.8 *µ*M, respectively. They inhibited HDAC enzyme in HCT116 cells at an IC_50_ value of 181.7 ± 22.04 and 70.2 ± 4.3 *µ*M, respectively, and inhibited human HDAC8 and 1 enzyme at an IC_50_ value of <50 *µ*M with QMJ-5 having greater specificity towards HDAC8. A reduction in the expression of HDAC8 and an increase in acetyl H3K9 expression were observed with the synthesized analogues. Both QMJ-2 and QMJ-5 treatment induced apoptosis through the activation of caspase 3/7 evident from 55.70% and 83.55% apoptotic cells, respectively. *In vivo* studies revealed a significant decrease in colon weight to length ratio in QMJ-2 and QMJ-5 treatment groups compared to DMH control. Furthermore, a reduction in aberrant crypt foci formation was observed in the treatment groups. The present study demonstrated the potential of novel 3-hydroxyflavone analogues as HDAC8 inhibitors with anticancer property against colorectal cancer.

## 1. Introduction

Cancer is a multifactorial disease and the second largest cause of death globally [1]. Over the years, knowledge about the pathophysiology of cancer has increased radically owing to the emergence of drug resistance and through the identification of newer hallmarks of cancer. Genetic predisposition was thought be one of the prime concerns in understanding the etiological background of cancer; however, the treatment modalities were still not successful based on either biochemically mediated or targeting genetically predisposed factors. This has created an arousal of interest in the field of epigenetic research. Researchers are earnestly attempting to develop newer therapies for cancer to improve the quality of life of patients. To support the continuous efforts made in developing anticancer agents, epidemiological survey shows that the overall mortality rate caused due to cancer has decreased by 25% from 1991 to 2014 [2].

Cancer is categorized into various types among which colorectal cancer (CRC) is the third most lethal malignancy diagnosed worldwide. The incidence of CRC is anticipated to increase by 60% globally with a mortality of 1.1 million by 2030 [3]. CRC is classified into four different stages, and the survival rate is solely dependent on the stage of diagnosis [4]. Patients who are diagnosed in the early stages (Stages I and II) have a 5-year survival rate of 90%, whereas patients who are diagnosed in the late stages (Stages III and IV) of CRC have a survival rate of 13.1% [5]. There are numerous risk factors associated with CRC that includes familial history, inherited genetic mutations, and lifestyle factors [6]. It is also noteworthy to mention the role of epigenetics in CRC. Studies have shown that epigenetic alterations play a crucial role in the transformation of normal colon epithelial cells into adenomatous polyps [7]. The epigenetic enzyme histone deacetylase (HDAC) has emerged out as an important regulator in the maturation and transformation of colon cells with several HDACs being upregulated in colon tumors [8]. Among the HDAC enzymes, expression of HDAC8 is highly prevalent in colon cancer where its expression level is higher compared to healthy tissues [9]. Studies have shown that knockdown of HDAC8 inhibits the proliferation of various cancer cells including colon cancer cells [10]. Although HDAC8 belongs to class I HDACs, unlike other members, HDAC8 functions as a deacetylase without being a part of multiprotein repressive complex making it a druggable target. Since the currently available chemotherapeutic agents for CRC has their own limitations, there is a scope of developing new drug candidates which could act as an alternative from the existing therapy.

Natural products are one of the most efficient and productive sources of lead molecules for the development of novel drugs. Among them, chalcones and polyphenols are some of the most widely studied molecules as anticancer agents [11]. Flavonoids, a distinct class of polyphenolic compounds ubiquitously present in plants, serve as phytonutrients and protect us against various forms of cancer [12]. Among the flavonoids, quercetin, a naturally occurring polyhydroxylated flavonol, has been found to exhibit broad spectrum of biological activities [13]. The presence of C2=C3 double bond and the 3-OH group in quercetin is highly decisive for its antitumor properties [14]. Studies have reported the potential of quercetin in eliciting epigenetic changes through the alteration of DNA methylation and histone acetylation levels [15]. However, the ability of quercetin in inhibiting HDAC8 in colon cancer has not been reported. Since flavonols have less adverse effect than chemotherapeutic drugs, they could provide better therapeutic options. Thus, in the present study, the polyphenol quercetin and a series of synthetic 3-hydroxyflavone analogues were evaluated for their *in vitro* HDAC8 inhibition and anticancer potential along with *in vivo* antitumor effects against colorectal cancer.

## 2. Materials and Methods

### 2.1. Chemicals and Instruments

Starting materials utilized in the synthesis of 3-hydroxyflavone analogues were procured from Sigma-Aldrich Co. LLC, St. Louis, MO, USA; Spectrochem Pvt. Ltd., Mumbai, MH, India; Merck Specialities Pvt. Ltd, Mumbai, MH, India; and TCI Co. Ltd., Tokyo, Japan. Dulbecco's Modified Eagle's Medium (DMEM), phosphatase inhibitor cocktail, protease inhibitor cocktail, Nonidet-P 40 (NP-40), BOC-Ac-Lys-AMC substrate, SAHA, and cell lysis buffer were obtained from Sigma-Aldrich Co. LLC, St. Louis, MO, USA. HDAC1 and HDAC6 enzymes were procured from Enzo Life Sciences, Inc. USA. Fetal bovine serum (FBS) was procured from Invitrogen BioServices Pvt. Ltd., Bangalore, India. 5-Fluorouracil was procured from Biochem Pharmaceutical Industries Ltd., Mumbai, India. 1,2-Dimethylhydrazine (DMH) was procured from TCI Chemicals (India) Pvt. Ltd. Buffers and chemicals for western blotting were obtained from Bio-Rad Laboratories Inc., Hercules, CA, USA. Primary antibodies, HDAC8 was obtained from Santa Cruz Biotechnology Inc. USA; p21^Waf1/Cip1^ was obtained from Cell Signaling Technology Inc., Danvers, USA; acetyl histone H3[Ac-Lys^9^] and GAPDH were obtained from Sigma-Aldrich Co. LLC, USA. Determination of the melting point was achieved using the capillary melting point apparatus from Toshniwal Systems and Instruments Pvt. Ltd., Chennai, TN, India. Thin layer chromatography was performed on precoated silica gel plates (Merck #60F254) and was visualized using a UV light source (254 or 366 nm) and/or iodine vapor. IR spectrophotometer (FTIR-8300, Shimadzu Co., Kyoto, Japan) was used to record the IR spectra utilizing KBr pellets. LC-MS (ESI) (LCMS-210A, Shimadzu Co., Kyoto, Japan) was used to record the mass spectra. ^1^H and ^13^C NMR spectra were recorded at 400 MHz (Ascend 400, Bruker Biosciences Corporation, Billerica, MA, USA) utilizing DMSO (D6) as a solvent. *δ* values (ppm) were used to report the chemical shifts and the signal multiplicities were represented by s (singlet), d (doublet), t (triplet), and m (multiplet).

### 2.2. Synthesis

#### 2.2.1. General Procedure for the Synthesis of (2E)-1-(2-Hydroxyphenyl)-3-(4-methylphenyl)prop-2-en-1-ones

Synthesis of chalcone intermediates was carried out using a previously described method with modifications [16, 17]. In brief, a solution of 2-hydroxyacetophenone (5 mM) in 10 ml methanol was mixed with 40% w/v aqueous potassium hydroxide (KOH) (10 ml), and the reaction was stirred for 20 min. To this reaction mixture, 4-methylbenzaldehyde (5 mM) was added and the reaction was stirred for 12 h or till the completion of a starting material. Furthermore, the mixture was poured on ice cold water and was neutralized using concentrated hydrochloric acid to obtain solid precipitate. The precipitate was then filtered, washed with ice cold water, and dried. The dried product was recrystallized from methanol to obtain the desired chalcone.

#### 2.2.2. General Procedure for the Synthesis of 3-Hydroxy-2-(4-methylphenyl)-4H-1-benzopyran-4-ones

**Figure 1:**
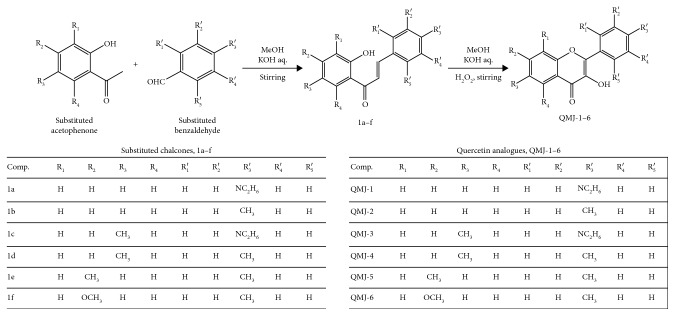
Synthesis of 3-hydroxyflavone analogues was carried out by the reaction of various substituted acetophenones with substituted benzaldehyde using 40% KOH and methanol to form chalcones (1a–f). The chalcones were cyclized using 10% KOH, methanol, and 15% H_2_O_2_ to produce 3-hydroxyflavones (QMJ-1–6).

Cyclization of chalcones into 3-hydroxyflavones was carried out using a previously described method with modifications [16, 17]. In brief, the chalcone intermediate (1 mM) was added in 5 ml of methanol to which 10% w/v aqueous potassium hydroxide (5 ml) was added and stirred for 20 min under ice cold condition. Next, 15% v/v hydrogen peroxide (H_2_O_2_) (2 ml) was added carefully into the reaction mixture, and stirring was continued for another 12 h. The reaction mixture was then poured on to ice cold water and neutralized using concentrated hydrochloric acid to obtain the final precipitate. It was then filtered, washed with ice cold water, and dried. The precipitate was recrystallized from methanol to obtain the final product. The synthetic scheme is shown in Figure 1.

### 2.3. Physical Parameters and Spectral Data for the Synthesized Analogues

#### 2.3.1. QMJ-1: 2-[4(Dimethylamino)phenyl]-3-hydroxy-4H-1-benzopyran-4-one

Yield = 76%, m. p. 270 ± 1°C uncorrected; IR (KBr): 3305.99 (-OH, Str), 1606.7 (C=O, Str), 1118.71 (C-O-C, Str), and 1070.4 (N-C, Str) cm^−1^; ^1^H NMR (DMSO-d6); *δ* = 3.022 (s, 6H, N-C2H6), 6.842 (d, 2H, Ar), 7.425 (t, 1H, Ar), 7.716 (m, 2H, Ar), 8.081 (m, 3H, Ar), and 9.188 (s, 1H, -OH) ppm. ^13^C NMR (DMSO-d6); *δ* = 111.88, 118.39, 118.61, 121.91, 124.77, 125.09, 129.43, 133.56, 137.73, 147.28, 151.50, 154.74, and 172.41 ppm. MS: *m/z* 281.

#### 2.3.2. QMJ-2: 3-Hydroxy-2-(4-methylphenyl)-4H-1-benzopyran-4-one

Yield = 72%, m. p. 250 ± 1°C uncorrected; IR (KBr): 3284.77 (-OH, Str), 1610.56 (C=O, Str), 1116.78 (C-O-C, Str), and 2916.37 (C-H, Str) cm^−1^; ^1^H NMR (DMSO-d6); *δ* = 2.395 (s, 3H, -CH3), 7.374 (t, 2H, Ar), 7.473 (d, 1H, Ar), 7.751 (m, 2H, Ar), 8.114 (t, 3H, Ar), and 9.555 (s, 1H, -OH) ppm; ^13^C NMR (DMSO-d6); *δ* = 21.52, 145.89, 139.24, 173.3, 124.99, 125.24, 134.11, 118.86, 154.99, 121.78, 128.98, 128.05, 129.61, and 140.27 ppm. MS: *m/z* 251.

#### 2.3.3. QMJ-3: 2-[4-(Dimethylamino)phenyl]-3-hydroxy-6-methyl-4H-1-benzopyran-4-one

Yield = 63%, m. p. 206 ± 2°C uncorrected; IR (KBr); 3269.34 (-OH, Str), 1600.92 (C=O, Str), 1168.86 (C-O-C, Str), 1058.92 (N-C, Str), and 2900.94 (C-H, Str) cm^−1^; ^1^H NMR (DMSO-d6); *δ* = 2.438 (d, 3H, -CH3), 3.017 (s, 6H, N-C2H6), 6.836 (d, 2H, Ar), 7.582 (d, 2H, Ar), 7.868 (s, 1H, Ar), 8.105 (d, 2H, Ar), and 9.106 (s, 1H, -OH) ppm. ^13^C NMR (DMSO-d6); *δ* = 111.87, 118.43, 121.60, 124.25, 129.37, 134.19, 134.78, 137.69, 147.10, 151.45, 153.09, and 172.33 ppm. MS (ESI): *m/z* (M−1) 294.00.

#### 2.3.4. QMJ-4: 3-Hydroxy-6-methyl-2-(4-methylphenyl)-4H-1-benzopyran-4-one

Yield = 61%, m. p. 182 ± 1°C uncorrected; IR (KBr); 3255.84 (-OH, Str), 1608.63 (C=O, Str), 1174.65 (C-O-C, Str), and 2918.30 (C-H, Str) cm^−1^; ^1^H NMR (DMSO-d6); *δ* = 2.395 (s, 3H, -CH3), 2.450 (s, 3H, -CH3), 7.372 (d, 2H, Ar), 7.630 (d, 2H, Ar), 7.900 (s, 1H, Ar), 8.129 (d, 2H, Ar), and 9.480 (s, 1H, -OH) ppm. ^13^C NMR (DMSO-d6); 20.91, 21.51, 118.68, 121.50, 124.31, 128.02, 129.06, 129.62, 134.50, 135.40, 139.18, 140.21, 145.75, 153.37, and 173.24 ppm. MS (ESI): *m/z* (M−1) 265.

#### 2.3.5. QMJ-5: 3-Hydroxy-7-methyl-2-(4-methylphenyl)-4H-1-benzopyran-4-one

Yield = 81%, m. p. 145 ± 1°C uncorrected; IR (KBr); 3261.63 (-OH, Str), 1610.56 (C=O, Str), 1166.93 (C-O-C, Str), and 2918.30 (C-H, Str) cm^−1^; ^1^H NMR (DMSO-d6); *δ* = 2.394 (s, 3H, -CH3), 2.481 (s, 3H, -CH3), 7.286 (d, 1H, Ar), 7.373 (d, 2H, Ar), 7.576 (s, 1H, Ar), 7.986 (d, 1H, Ar), 8.110 (d, 2H, Ar), and 9.449 (s, 1H, -OH) ppm; ^13^C NMR (DMSO-d6); *δ* = 21.51, 21.76, 145.46, 140.14, 173.17, 125.05, 126.55, 139.08, 118.33, 155.13, 119.58, 129.07, 127.95, 129.62, and 145.04 ppm. MS (ESI): *m/z* (M−1) 265.25.

#### 2.3.6. QMJ-6: 3-Hydroxy-7-methoxy-2-(4-methylphenyl)-4H-1-benzopyran-4-one

Yield = 72%, m. p. 195 ± 1°C uncorrected; IR (KBr); 3277.06 (-OH, Str), 1604.77 (C=O, Str), 1166.93 (C-O-C, Str), 2845.00 (-OCH_3_, Str), and 2972.31 (C-H, Str) cm^−1^; ^1^H NMR (DMSO-d6); *δ* = 2.400 (s, 3H, -CH3), 3.926 (s, 3H, -OCH3), 7.043 (s, 1H, Ar), 7.063 (s, 1H, Ar), 7.374 (d, 2H, Ar), 8.002 (s, 1H, Ar), 8.150 (d, 2H, Ar), and 9.437 (s, 1H, -OH) ppm. ^13^C NMR (DMSO-d6); *δ* = 21.49, 56.55, 100.74, 115.11, 115.63, 126.60, 127.79, 129.09, 129.58, 138.94, 139.97, 145.17, 156.93, 164.10, and 172.76 ppm. MS (ESI): *m/z* (M+1) 283.17.

### 2.4. *In Vitro* Studies

#### 2.4.1. Cell Lines and Their Maintenance

Human colorectal carcinoma cell line (HCT116) and African green monkey kidney epithelial cells (VERO) were obtained from National Centre for Cell Science, Pune, MH, India. For their maintenance, the cells were grown in Dulbecco's Modified Eagle's Medium (DMEM) which was supplemented with 10% fetal bovine serum (FBS) and 1 × penicillin/streptomycin at 37°C in CO_2_ incubator (NU-5501E/G, NuAire Inc., Plymouth, MN, USA) in a humidified atmosphere of 5% CO_2_ and 95% air.

#### 2.4.2. Cytotoxicity Study

Cytotoxicity for the synthesized analogues was determined using MTT assay [18]. In brief, HCT116 and VERO cells were harvested from the tissue culture flask and seeded in sterile 96 well plates at a density of 5000 cells per well. After overnight adherence, different concentrations of test compounds were added and incubated for 48 h. Next, 50 *µ*l of the MTT reagent (HiMedia Laboratories, Mumbai, India) (2 mg/ml in the sterile phosphate buffer saline (PBS)) was added into each well and incubated for 3 h. The formazan crystals formed were solubilized using 100% dimethyl sulfoxide (DMSO), and the optical density was measured at 540 nm using the microplate reader (ELx800, BioTek Instruments Inc., Winooski, VT, USA).

#### 2.4.3. Whole Cell HDAC Inhibition Assay

Confluent HCT116 cells were harvested and seeded in 96 well sterile black well plates at a density of 2000 cells per well and incubated overnight. Furthermore, the cells were treated with different concentrations of test compounds for a period of 18 h followed by the addition of 15 mM BOC-Ac-Lys-AMC substrate and incubated for 1 h. The reaction was then terminated by adding 50 *µ*l of stop solution (trypsin 2 mg/ml, 1% NP40, and 1 *µ*l SAHA) in the HDAC assay buffer (25 mM Tris-HCl (pH 8.0), 137 mM NaCl, 2.7 mM KCl, and 1 mM MgCl_2_). The reaction was then allowed to proceed for 15 min at 37°C. Finally, the fluorescence intensity was measured at 360 nm excitation and 460 nm emission using a fluorescence microplate reader (FLx800, BioTek Instruments Inc., Winooski, VT, USA) [19].

#### 2.4.4. HDAC8 Enzyme Inhibition Assay

The enzymatic reaction was conducted as per Enzo Life Science, NY, USA (BML-AK518) kit protocol. In brief, 10 *µ*l of different concentration of test compounds were added to a 40 *µ*l reaction mixture containing the HDAC assay buffer, HDAC8 substrate, and recombinant human HDAC8 enzyme. The reactions were incubated for 20 min at 30°C. Then, 50 *µ*l of the HDAC developer was added in each well, and the plate was incubated at room temperature for an additional 45 min. Fluorescence intensity was measured at an excitation of 360 nm and an emission of 460 nm using a fluorescence microplate reader (FLx800, BioTek Instruments Inc., Winooski, VT, USA).

#### 2.4.5. HDAC1 and 6 Enzyme Inhibition Assays

The HDAC1 and 6 assays were carried out with few modifications from the previously described method [20]. In brief, 10 *µ*l of different concentration of test compounds was added to 40 *µ*l of reaction mixture containing HDAC assay buffer, HDAC substrate, and recombinant human HDAC1 and 6 enzymes. The reactions were incubated for 30 min at 37°C. Then, 50 *µ*l of trypsin stop solution was added in each well, and the plate was incubated at room temperature for an additional 20 min. Fluorescence intensity was measured at an excitation of 360 nm and an emission of 460 nm using a fluorescence microplate reader (FLx800, BioTek Instruments Inc. Winooski, VT, USA).

#### 2.4.6. Western Blotting

After various treatments for 24 h, cells were lysed, and protein quantification of the lysates was estimated using the BCA™ kit (Thermo Fisher Scientific Inc., Waltham, MA, USA). Proteins samples (30 *µ*g) were resolved on a 10% SDS-PAGE and transferred onto a PVDF membrane and blocked with 5% nonfat milk in TBST (tris-buffered saline with Tween-20) buffer for 1 h followed by washing in TBST buffer. The membrane was then probed with primary antibodies (GAPDH, HDAC8, p21^Waf1/Cip1^, and acetyl-histone H3[Ac-Lys^9^]) and incubated overnight at 4°C. The membrane was washed with TBST buffer and incubated with horseradish peroxidase-conjugated secondary antibody for 1 h at room temperature. Signal was captured using SuperSignal™ West Pico Plus chemiluminescent substrate (Thermo Fischer Scientific Inc., Waltham, MA, USA) on gel documentation system (G : BOX Chemi XRQ, Syngene). The protein band densities were estimated using the software ImageJ (version 1.46r, NIH, Bethesda, MD, USA) [21].

#### 2.4.7. Annexin V Staining

Mechanism of cell death induced by synthesized analogues was determined using annexin V staining. In brief, as per annexin V flow cytometry kit protocol (#MCH100105), 1 × 10^6^ cells were seeded in a cell culture dish and after overnight adherence, were incubated with test compounds. Cells were then detached by trypsinization, centrifuged, and washed with PBS. Cell suspension (100 *µ*l) was mixed with Muse annexin V reagent (100 *µ*l) and incubated for 20 min at room temperature. The stained cells were quantitatively analyzed for live, early, and late apoptosis using Muse cell analyzer (#0500-3115 Merck Millipore).

#### 2.4.8. Caspase 3/7 Activation Assay

The downstream signaling pathway of apoptosis was determined using the caspase 3/7 activation assay. In brief, as per the caspase 3/7 activation kit protocol (#MCH100108), 1 × 10^6^ cells were seeded in the cell culture dish and after overnight adherence, were incubated with the test compounds. The cells were detached by trypsinization, centrifuged, and washed with PBS. Cell suspension (50 *µ*l) was mixed with the caspase 3/7 antibody reagent (5 *µ*l) and incubated at 37°C temperature for 30 min. 7-AAD (7-amino-actinomycin D) working solution (150 *µ*l) was added, and stained cells were then analyzed in Muse Cell Analyzer (#0500-3115 Merck Millipore).

#### 2.4.9. Cell Cycle Analysis

Cell cycle analysis was performed to estimate the effect of synthesized analogues on the different phases of the cell cycle. In brief, 1 × 10^6^ cells were seeded in the cell culture dish and allowed to attach overnight followed by incubation with test compounds. After 48 h, the cells were detached by trypsinization, centrifuged, and washed with PBS. The cell pellets were fixed in 70% v/v ice cold ethanol and stored at −20°C for 24 h. After fixing, the pellets were dislodged in PBS and stained with propidium iodide solution and analyzed using Accuri C6 flow cytometer (BD Biosciences, San Jose, CA, USA) [22].

### 2.5. *In Vivo* Studies

#### 2.5.1. Animals

Animal care and handling were carried out according to the guidelines of the Committee for the Purpose of Control and Supervision of Experiments on Animals (CPCSEA), after seeking the approval of the research proposal by institutional animal ethics committee (IAEC) (IAEC/KMC/23/2015). Male Wistar rats inbred at the central animal research facility (CARF), Manipal academy of higher education (MAHE), was used in the present study. The experimental room had a temperature of 23 ± 2°C and a humidity of 50 ± 5%. The animals were housed in sterile polypropylene cages containing sterile paddy husk and were provided with 12 h light and dark cycles.

#### 2.5.2. Acute Toxicity Studies

The safe dose of the promising two analogues (QMJ-2 and QMJ-5) and quercetin were evaluated using Organization for Economic Cooperation and Development (OECD), 425 guidelines. Limit test was performed using 2000 mg/kg dose. Animals were observed for the first 4 h continuously and then monitored each day for a period of 14 days.

#### 2.5.3. Preparation of the Test Compounds and Standard Drug

The two test compounds (QMJ-2 and QMJ-5) and quercetin were suspended in 0.25% w/v sodium carboxymethyl cellulose (CMC) and were administered orally (*p.o.*). 5-Fluorouracil (5-FU) which was used as a standard was procured in the form of injection and was administered intraperitonially (*i.p.*).

#### 2.5.4. 1,2-Dimethylhydrazine-(DMH-) Induced Colon Cancer in Wistar Rats

Colon cancer in experimental animals was induced using the highly specific carcinogen DMH, with few modifications from the previously described procedure [23]. DMH was administered at a dose of 30 mg/kg *i.p.* once a week for 20 weeks. The incidence of aberrant crypt foci (ACF) and adenocarcinoma were confirmed by sacrificing few animals after 20 weeks of induction with DMH. Furthermore, the animals were randomized into five experimental groups, and various treatments were administered for a period of 21 days followed by the assessment of different parameters in the experimental animals.

*(1) Experimental Groups*

  Group 1: animals received 0.25% CMC in water *p.o.*
  Group 2: animals received 0.25% CMC in water *p.o.* (DMH control)
  Group 3: animals received 5-FU at 10 mg/kg *i.p.*
  Group 4: animals received quercetin at 100 mg/kg *p.o.*
  Group 5: animals received QMJ-2 at 100 mg/kg *p.o.*
  Group 6: animals received QMJ-5 at 100 mg/kg *p.o.*

*(2) ACF Formation and Adenocarcinoma Incidence*. The entire colon was excised from the experimental animals and was used to study the incidence of adenocarcinoma. The count and size were noted from each animal. For ACF formation, the distal part of the colon was excised, and the tissue was then cut open and fixed with 10% buffered formalin for 12 h. Following this, the tissue was stained with 0.1% methylene blue in PBS for 5 min. The tissues were then observed under microscope for ACF formation and were calculated as the number of counts/5 cm^2^ in colon tissue.

*(3) Colon Weight/Length Ratio and Organ Index*. Length of the excised colon was measured in centimeters, and weight was measured in g. Colon weight/length ratio was then calculated. For the determination of organ index, major organs including spleen, liver, and kidney of the experimental animals were weighed in g and the respective index was calculated.

*(4) Histopathology of Colon*. Colon tissue was collected at the end of the study period from different groups and stored in 10% neutralized buffered formalin and processed for histopathological changes. The stained slides were subsequently analyzed under microscope for anatomical changes.

### 2.6. Statistical Analysis

Experimental data were analyzed using one-way ANOVA followed by Tukey's multiple comparison tests using Prism 5.03 (Graph Pad Software Inc., La Jolla, CA, USA). All values are expressed as mean ± SEM of 6 animals. Significance was considered at *p* < 0.05.

## 3. Results

### 3.1. *In Vitro* Studies

#### 3.1.1. Cytotoxicity Study

Cytotoxic potential of the synthesized analogues was estimated using MTT assay. QMJ-1, QMJ-2, QMJ-3, QMJ-5, and QMJ-6 were found to be cytotoxic against colon cancer cells after 48 h of treatment. Among them, QMJ-2 and QMJ-5 were most cytotoxic with an IC_50_ value of 68 ± 2.3 and 27.4 ± 1.8 *µ*M, respectively. However, in Vero cells, the IC_50_ values of QMJ-2 and QMJ-5 was found to be 140 ± 6.8 and 55.6 ± 3.8 *µ*M.  Table 1 shows the IC_50_ value of the synthesized analogues on both cell lines.

#### 3.1.2. Whole Cell HDAC Inhibition Assay

The ability of the synthesized analogues to inhibit the epigenetic enzyme HDAC was estimated using whole cell HDAC enzyme inhibition assay in HCT116 cells. Dose-dependent enzyme inhibition was observed in QMJ-2- and QMJ-5-treated cells with an IC_50_ value of 181.7 ± 22.04 and 70.2 ± 4.3 *µ*M, respectively. However, the other analogues were less efficacious with higher IC_50_ values. The nonspecific HDAC inhibitor SAHA was found to have an IC_50_ value of 1.2 ± 0.1 *µ*M.  Table 2 shows the IC_50_ values obtained from this assay.

#### 3.1.3. HDAC8, 1, and 6 Enzyme Inhibition Assays

The test compounds were evaluated for their isoform-specific HDAC enzyme inhibition. QMJ-2 and QMJ-5 were found to be specific toward class I HDAC enzyme with an IC_50_ value of 34.11 and 47.7 *µ*M, respectively, against HDAC1 enzyme and 36.03 and 24.0 *µ*M, respectively, against HDAC8 enzyme. The IC_50_ value of QMJ-2 and QMJ-5 was found to be 165.1 and 163.35 *µ*M, respectively, against HDAC6 enzyme. Quercetin inhibited HDAC1 and HDAC8 with an IC_50_ value of 26.72 and 15.4 *µ*M, respectively, and inhibited HDAC6 at an IC_50_ value of 43.39 *µ*M. The nonspecific HDAC inhibitor SAHA had an IC_50_ value of 0.026 and 0.7 *µ*M against HDAC1 and HDAC8 enzyme, respectively, whereas 0.098 *µ*M against HDAC6 enzyme.  Table 3 shows the results of HDAC8, 1, and 6 enzyme inhibition assays.

#### 3.1.4. Western Blotting Analysis

**Figure 2:**
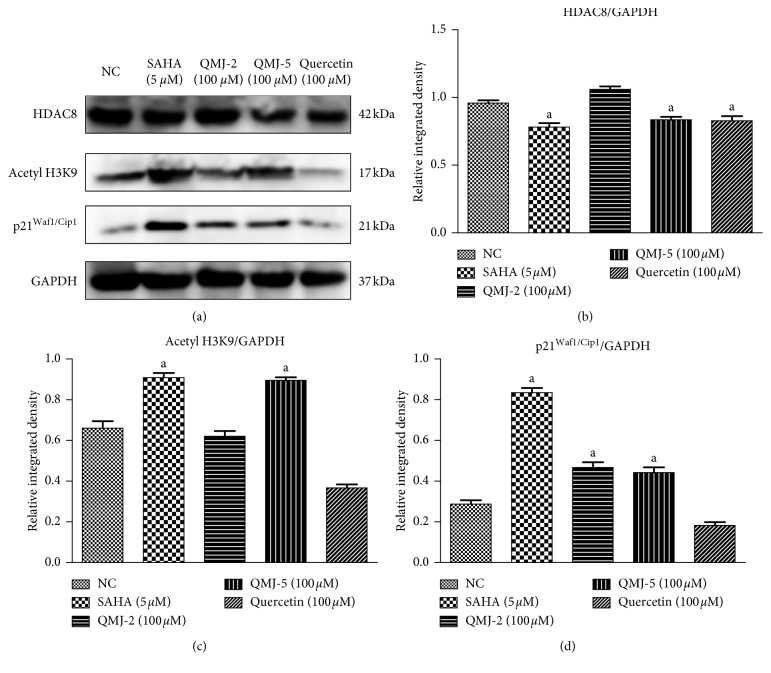
Effect of various compounds incubated for 24 h on HDAC8, acetyl histone H3K9, and p21^Waf1/Cip1^ protein expression in HCT116 cells. All values are expressed as mean ± SEM of three experiments. ^a^*p* < 0.05 vs. normal control (NC).

Protein expression studies indicated a significant reduction in HDAC8 expression in SAHA-, QMJ-5-, and quercetin-treated groups compared to normal. Moreover, a significant increase in the expression of acetylated histone H3K9 was observed in SAHA- and QMJ-5-treated groups compared to normal control. The expression of the cell cycle regulatory protein p21^Waf1/Cip1^ was also increased in SAHA-, QMJ-2-, and QMJ-5-treated groups which were significant compared to normal control.  Figure 2 shows the expression of target proteins in various treatment groups along with their relative density.

#### 3.1.5. Annexin V Staining

**Figure 3:**
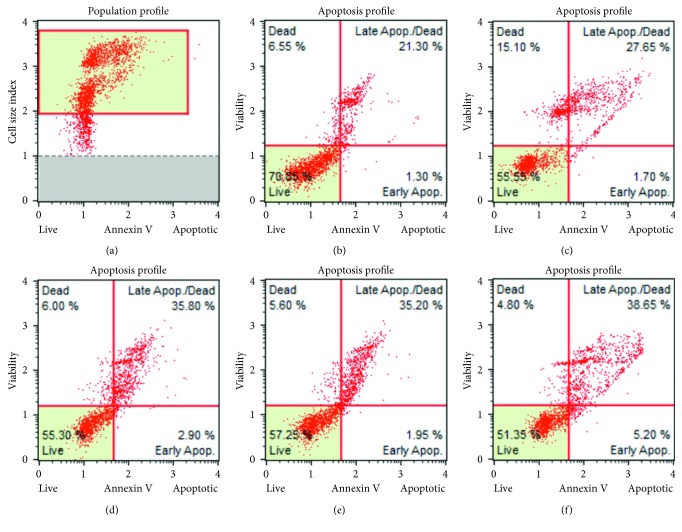
Apoptotic profile after treatment with various compounds in HCT116 cells was determined as % live,% early, and late apoptosis and % dead cells by MUSE cell analyzer: (a) normal control; (b) normal control; (c) SAHA (5 *µ*M); (d) quercetin (100 *µ*M); (e) QMJ-2 (100 *µ*M); (f) QMJ-5 (50 *µ*M).

Induction of apoptosis was determined by annexin V staining in HCT116 cells. It was observed that the percentage of live cells in the normal control group was 70.85%, whereas treatment with QMJ-2 and QMJ-5 decreased the percentage of live cells drastically to 57.25% and 51.35%, respectively. Furthermore, it was found that the percentage of cells undergoing late apoptosis was 35.20% and 38.65% in QMJ-2 and QMJ-5 groups, respectively, compared to that of 21.30% in the normal control group. Percentage of cells undergoing apoptosis both in SAHA- and in quercetin-treated groups were found to be 27.65% and 35.80%, respectively.  Figure 3 shows the apoptotic profile of various treatment groups.

#### 3.1.6. Caspase 3/7 Activation Assay

**Figure 4:**
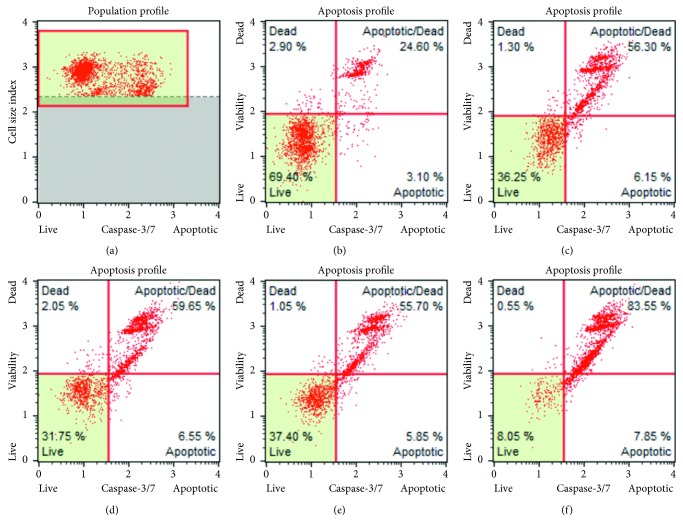
Apoptosis induction through the activation of caspase 3/7 after treatment with various compounds in HCT116 cells was determined as % live, % early, and late apoptosis and % dead cells by the MUSE cell analyzer. (a) Normal control; (b) normal control; (c) SAHA (5 *μ*M); (d) quercetin (100 *μ*M); (e) QMJ-2 (100 *μ*M); (f) QMJ-5 (50 *μ*M).

The activation of executioner caspase 3/7 was determined in HCT116 cells. It was found that QMJ-2- and QMJ-5-treated cells activated apoptosis through the induction of caspase 3/7. The percentage of live cells was found to be 37.40% and 8.05% in QMJ-2- and QMJ-5-treated groups when compared to 69.40% in normal control. An increase in the percentage of apoptotic cells was observed in QMJ-2- and QMJ-5-treated groups and was found to be 55.70% and 83.55%, respectively, when compared to that of 24.60% in the normal control group. Furthermore, percentage of cells undergoing apoptosis in both SAHA- and quercetin-treated group was found to be 56.30% and 59.65%, respectively.  Figure 4 shows the results from the caspase 3/7 activation assay.

#### 3.1.7. Cell Cycle Analysis

**Figure 5:**
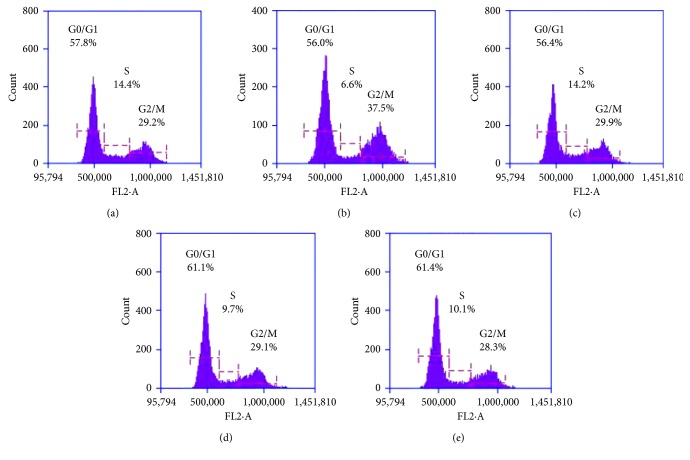
Effect of various compounds on different phases of HCT116 cells was estimated using flow cytometer. Histogram plots display the different phases of the cell cycle and the percentage of cells in each phase. (a) Normal control; (b) SAHA (5 *μ*M); (c) quercetin (100 *μ*M); (d) QMJ-2 (100 *μ*M); (e) QMJ-5 (50 *μ*M).

In normal control group, the percentage of cells in G_0_/G_1_, S, and G_2_/M phase was found to be 57.8%, 14.4%, and 29.2%, respectively. Treatment with QMJ-2 and QMJ-5 caused an increase in the percentage of cells in G_0_/G_1_ phase. Treatment with SAHA arrested G_2_/M phase (37.5%) of the cell cycle.  Figure 5 shows the histogram plot of the cell cycle in various treatment groups.

### 3.2. *In Vivo* Studies

#### 3.2.1. Toxicity Study

Both QMJ-2 and QMJ-5 along with quercetin were found to be safe and well tolerated at a dose of 2000 mg/kg *p.o*. There was no mortality observed in the experimental animals at the tested dose. Further studies were carried out using 1/20^th^ of the administered dose.

#### 3.2.2. ACF Formation and Adenocarcinoma Incidence

The incidence of colorectal cancer is detected through the formation of ACF in colonic mucosa. In the present study, ACF formation was observed in all the groups that had been administered with the toxicant DMH. ACF count of the excised colon in DMH control, 5-FU-, quercetin-, QMJ-2- and QMJ-5-treated groups were found to be 25 ± 2.1, 10 ± 1.3, 19 ± 2.6, 16 ± 2.1, and 15 ± 2.6, respectively. A significant (*p* < 0.05) reduction in the ACF count was observed in 5-FU-, QMJ-2- and QMJ-5-treated groups when compared to that of the DMH control group. Incidence of colon adenocarcinoma was observed in all the treatment groups, whereas a significant (*p* < 0.05) reduction in adenocarcinoma was found only in 5-FU- and QMJ-5-treated groups.  Table 4 shows the results of ACF formation and adenocarcinoma incidence in various treatment groups.

#### 3.2.3. Colon Weight/Length Ratio and Organ Index

**Figure 6:**
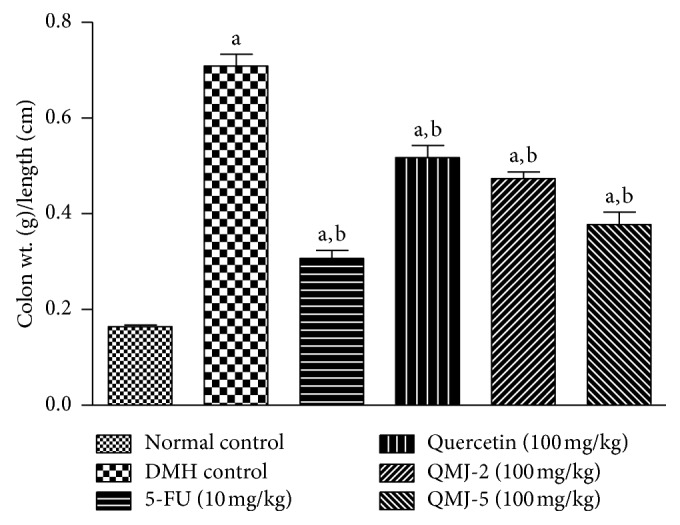
The bar graphs represent colon weight/colon length ratio. All values are expressed as mean ± SEM of six animals. ^a^*p* < 0.05 vs. normal control; ^b^*p* < 0.05 vs. DMH control.

**Figure 7:**
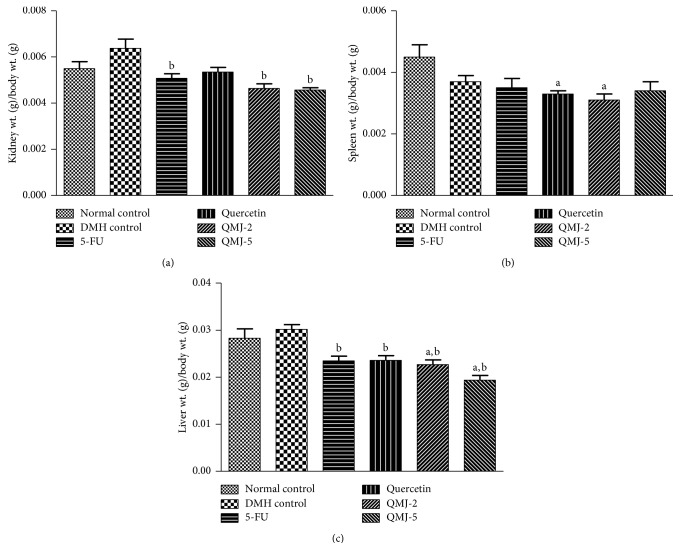
The bar graphs represent the effect of various treatments on the major organ index. All values are expressed as mean ± SEM of six animals. ^a^*p* < 0.05 vs. normal control; ^b^*p* < 0.05 vs. DMH control. (a) Kidney index; (b) spleen index; (c) liver index.

A significant (*p* < 0.05) increase in colon weight/length ratio was observed in DMH control when compared with the normal control group. Treatment with 5-FU and other test compounds significantly (*p* < 0.05) reduced the colon weight/length ratio when compared with DMH control. Among the test compounds, QMJ-5 was comparatively better in reducing colon weight/length ratio compared to other groups (Figure 6). An increase in the kidney index was observed in the DMH control group which was significantly reduced in 5-FU, QMJ-2 and QMJ-5 treatment groups. A significant decrease in the spleen index was observed in quercetin and QMJ-2 treatment groups compared to normal control. Significant reduction in the liver index was observed in QMJ-2 and QMJ-5 treatment groups when compared with the normal control and a significant reduction in 5-FU, quercetin, QMJ-2, and QMJ-5 was observed when compared with DMH control (Figure 7).

#### 3.2.4. Histopathology

**Figure 8:**
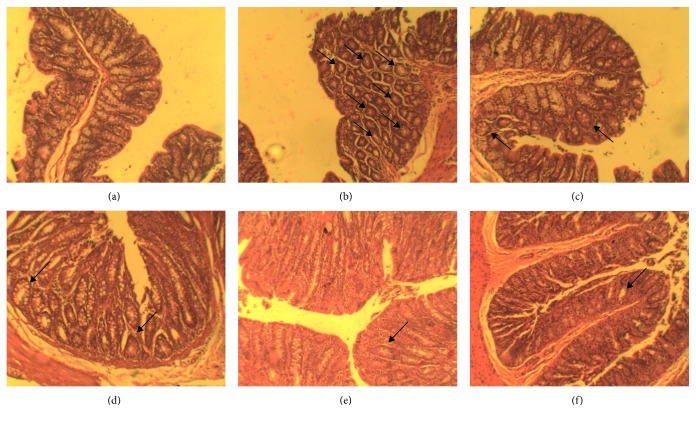
Histopathological changes of colon tissues from different treatment groups at 40x magnification. Arrows indicate aberrant crypt foci. (a) Normal control; (b) DMH control; (c) 5-FU; (d) quercetin; (e) QMJ-2; (f) QMJ-5.

Histopathological examination of the colon tissues in the normal control group showed the presence of villi without any signs of dysplasia or aberrant crypt. However, in the DMH control group, the presence of aberrant crypt was prominent with an abnormal architecture of the colonic mucosa. However, reduction in the aberrant crypt and a restoration towards the normal morphology of colon were observed in the treatment groups.  Figure 8 shows the representative histopathological image.

## 4. Discussion

Colorectal cancer (CRC) is one of the lethal forms of malignancy and is a global burden both in terms of morbidity and economic expenditure. Various factors are associated with the pathogenesis of CRC among which numerous studies have highlighted the role of epigenetics. The epigenetic enzyme HDACs are overexpressed in colorectal cancer leading to cellular proliferation and differentiation [24]. Among the various HDAC enzymes, HDAC8 is an emerging target in cancer research. Genomic analysis of colon cancer cells shows an increased expression of HDAC8 compared to normal cells [25]. Studies have reported that HDAC8 inhibits apoptosis in colon cancer cells by repressing Bcl-2-modifying factor (BMF) transcription [26]. Interestingly, the deacetylase activity of HDAC8 is exhibited by the enzyme without being associated with any multiprotein complex [27]. These features provide rationale for developing inhibitors against HDAC8 that could be beneficial towards CRC. Natural molecules, especially polyphenols, have been widely studied and are found to be valuable for the treatment of cancer. Among the diverse polyphenolic compounds, flavonols exhibit beneficial property in cancer. The structural feature of flavonols including C2=C3 unsaturation and the presence of the 3-hydroxy group is a crucial determinant for their antitumor property. Exploiting this feature, the polyphenol quercetin and a series of 3-hydroxyflavone analogues were evaluated for their HDAC8 inhibitory potential and anticancer property against colorectal cancer.

The screening of probable anticancer compounds is facilitated based on cytotoxic studies that are reliable and often used to determine their potential in inhibiting the growth of cancer cells [28]. MTT assay was used to determine cytotoxicity where it was found that, out of all the compounds synthesized and tested against colon cancer cells, two analogues QMJ-2 and QMJ-5 were found to be most promising. Furthermore, both the analogues exhibited cytotoxicity at two-fold lower concentration when compared to that of the inhibitory concentration observed in normal (Vero) cell line.

Studies have reported the role of epigenetic alterations in understanding the pathophysiology of colon cancer [29]. Among the epigenetic enzymes that are involved, HDACs play a crucial role in the development of colon cancer. In the present study, whole cell HDAC inhibition assay was performed in colon cancer cells. It was observed that, QMJ-2, QMJ-5, and quercetin had a dose-dependent inhibition on HDAC enzyme. Since both the analogues inhibited HDAC enzyme nonspecifically, we further explored their potential to inhibit human HDAC8, 1, and 6 enzymes. HDAC8 and 1 belong to class I, whereas HDAC6 belongs to class IIb superfamily of HDAC enzymes [30]. It was evident from our study that QMJ-2 and QMJ-5 inhibited HDAC8 and HDAC1 enzymes with an IC_50_ value of less than 50 *µ*M. Furthermore, both the compounds inhibited HDAC6 enzyme at a higher concentration suggesting their specificity towards class I HDACs. In addition, QMJ-5 inhibited HDAC8 enzyme at a lower concentration compared with HDAC1 enzyme suggesting its specificity towards HDAC8. The nonspecific HDAC inhibitor SAHA and the polyphenol quercetin also inhibited various HDAC enzymes as evident from our study. Although SAHA inhibited different HDAC isoforms at a lower concentration, it was not specific. Studies have reported that HDAC inhibitors repress histone deacetylation leading to the hyperacetylation of histone protein and several other genes [31]. In the present study, we observed a reduction in the expression levels of HDAC8 after treatment with the synthesized compound. Moreover, an increase in the acetylation levels of histone H3K9 was observed among the treatment groups, suggesting their ability to alter the chromatin state which would further lead to the expression of tumor suppressor genes.

To study the plausible mechanism of cell death induced by the analogues in cancer cells, apoptotic assay was performed. In the event of apoptosis, a membrane phospholipid, phosphatidylserine translocate from the inner leaflet of the cell membrane to the outer membrane of the cell [32]. Annexin V, a specific marker binds with phosphatidylserine and facilitates in the determination of apoptosis. It was found that, QMJ-2 and QMJ-5 were able to induce apoptosis in colon cancer cells evident from the percentage of cells in the early and late stages of apoptosis. Furthermore, to determine the downstream signaling pathway of apoptosis, activation of caspase 3/7 was assessed. Caspases are a family of cysteine protease that plays a primary role during apoptosis [33]. The executioner caspase (3, 6, and 7) conducts the mass proteolysis of target proteins eventually causing apoptosis. It was found that, the analogues activated caspase 3/7 leading to the induction of the mitochondrial pathway of apoptosis as the mechanism of cell death.

Cell cycle analysis is a useful tool that provides information on various phases (G_0_/G_1_, S, and G_2_/M) of cell cycle depending upon the DNA content. The initiation of cell cycle progression is controlled at the G_1_ phase where, any damage to the DNA would retard the progression and allow the cell to repair before it could enter the S phase [34]. However, in conditions where, there are no further signs of cellular repair, the mitochondrial pathway of apoptosis gets activated mediating through the accumulation of phosphorylated p53. In the present study, cell cycle analysis revealed that both QMJ-2 and QMJ-5 arrested the cell cycle of colon cancer cells at the G_0_/G_1_ phase. The cyclin-dependent kinase inhibitor p21 regulates cell cycle progression and plays an important role in the prevention of tumor development [35]. Studies have reported that HDAC inhibitors induce the expression of *p21*^*Waf1*^ gene leading to cell cycle arrest [36]. We observed an increase in the expression level of p21^Waf1/Cip1^ in SAHA, QMJ-2, and QMJ-5 treatment groups suggesting their ability to induce the expression of p21^Waf1/Cip1^ leading to cell cycle arrest.

Previous reports suggest that compounds with HDAC inhibitory potential induce apoptosis and arrest cell cycle through the mitochondrial pathway [37]. Moreover, studies have demonstrated that polyphenols induce cell cycle arrest and apoptosis through the inhibition of histone deacetylase enzyme [38]. Similar results were obtained from our study where QMJ-2 and QMJ-5 inhibited HDAC8 expression and increased the levels of acetylated histone H3K9. The compounds also arrested the cell cycle at the G_0_/G_1_ phase through the expression of p21^Waf1/Cip1^ protein and induced apoptosis through the activation of caspase 3/7.

Promising results from *in vitro* studies supported the need to assess the *in vivo* potential of the analogues in DMH-induced colorectal cancer in rats. DMH is an extremely precise colorectal carcinogen that along with its metabolite azoxymethane (AOM) hastens the development of colorectal cancer in rats [39]. The formation of aberrant crypt foci (ACF) is the initial lesion observed in colon tissues, and a reduction in the ACF count is an essential indicator of recovery from colorectal cancer [40]. In the present study, histopathological analysis indicated the formation of ACF and abnormal mucosal architecture in DMH control group. Histopathology of various treatment groups revealed the reduction in ACF formation and restoration of normal colon morphology. It was observed that both QMJ-2 and QMJ-5 significantly reduced the ACF formation compared with that of DMH control group, suggesting their role in protecting the colon mucosa. The incidence of adenocarcinoma is another key-determining factor for the development of colorectal cancer. A marked reduction in the adenocarcinoma formation was observed in the QMJ-5-treated group when compared with that of the DMH control group further supporting their protective effect. Administration of DMH causes alteration in the colonic mucosa and the formation of adenocarcinoma, leading to an increase in colon weight and shortening in the length of colon [41]. Similar results were obtained from our study where an increase in colon weight/length ratio was observed in the DMH control group compared to normal. QMJ-2 and QMJ-5 reduced the ratio which demonstrated their protective effect against colorectal cancer. Organ index studies exhibited that there was no significant change in the kidney index of QMJ-2 and QMJ-5 treatment groups compared to normal. However, there was a significant reduction in the liver index of animals treated with QMJ-2 and QMJ-5 suggesting further studies to determine the mechanism. Since the available HDAC inhibitors are effective against leukemia, newer HDAC inhibitors effective against solid tumors is the need of the hour. From the present study, it was evident that QMJ-2 and QMJ-5 inhibited HDAC enzyme *in vitro* and had a protective role against DMH-induced colon cancer *in vivo*.

## 5. Conclusion

The present study revealed that both analogues QMJ-2 and QMJ-5 and the polyphenol quercetin were found to be cytotoxic and inhibited HDAC enzyme, where QMJ-5 showed greater specificity towards HDAC8. Induction of apoptosis by QMJ-2 and QMJ-5 in colon cancer cells was mediated through the activation of caspase 3/7 along with the cell cycle arrest at the G_0_/G_1_ phase through the expression of p21^Waf1/Cip1^. The synthesized analogues reduced the formation of ACF and adenocarcinoma in the animal model of colorectal cancer. Thus, the present study identified the potentials of novel 3-hydroxyflavone analogues as HDAC8 inhibitors with anticancer property against colorectal cancer providing a lead for new drug development.

## Figures and Tables

**Table 1:** MTT assay.

Sl. no.	Compound	IC_50_ (*µ*M ± SEM)	
		HCT116	Vero
1	QMJ-1	102.5 ± 1.8	186.4 ± 4.9
2	QMJ-2	68 ± 2.3	140 ± 6.8
3	QMJ-3	70.3 ± 4.1	80.1 ± 3.7
4	QMJ-4	>400	>400
5	QMJ-5	27.4 ± 1.8	55.6 ± 3.8
6	QMJ-6	94 ± 2.1	153 ± 2.3
7	Quercetin	107.6 ± 1.2	201.5 ± 7.5
8	SAHA	3.1 ± 0.35	4.6 ± 1.6

All values are expressed as mean ± SEM of three experiments.

**Table 2:** Whole cell HDAC enzyme inhibition assay.

Sl. no.	Compound	HDAC inhibition IC_50_ (*µ*M ± SEM)
1	QMJ-1	1023.1 ± 5.2
2	QMJ-2	181.7 ± 22.04
3	QMJ-3	1124 ± 6.2
4	QMJ-4	1011.2 ± 3.4
5	QMJ-5	70.2 ± 4.3
6	QMJ-6	984.2 ± 2.5
7	Quercetin	167.8 ± 6.9
8	SAHA	1.2 ± 0.1

The values are expressed as mean ± SEM of three experiments.

**Table 3:** Isoform-specific HDAC enzyme inhibition assay.

Sl. no.	Compound	HDAC8	HDAC1	HDAC6
1	QMJ-2	36.03 ± 4.17	34.11 ± 3.83	165.1 ± 11.1
2	QMJ-5	24.00 ± 2.8	47.7 ± 6.3	163.35 ± 7.05
4	Quercetin	15.4 ± 1.53	26.72 ± 3.77	43.39 ± 2.17
3	SAHA	0.7 ± 0.025	0.026 ± 0.002	0.098 ± 0.012

The IC_50_ (*µ*M) values are expressed as mean ± SEM of three experiments.

**Table 4:** Effect of various treatments on the ACF count and adenocarcinoma incidence in colon tissue.

Sl. no.	Groups	ACF/5 cm^2^	Adenocarcinoma
1	Normal control	0	0
2	DMH control	25 ± 2.1	17 ± 1.7
3	5-FU	10 ± 1.3^a^	8 ± 1.1^a^
4	Quercetin	19 ± 2.6	15 ± 1.1
5	QMJ-2	16 ± 2.1^a^	14 ± 1.3
6	QMJ-5	15 ± 2.6^a^	11 ± 0.8^a^

All values are expressed as mean ± SEM of six animals. ^a^*p* < 0.05 vs. DMH control.

## Data Availability

The data used to support the findings of this study are available from the corresponding author upon request.
